# Ground truthing the cost of achieving the EAT lancet recommended diets: Evidence from rural India

**DOI:** 10.1016/j.gfs.2021.100498

**Published:** 2021-03

**Authors:** Soumya Gupta, Vidya Vemireddy, Dhiraj K. Singh, Prabhu Pingali

**Affiliations:** a Tata Cornell Institute for Agriculture and Nutrition, Cornell University, United States; b Senior Research Scientist, Institute for Research and Growth, India; c Indian Institute of Management Ahmedabad, India

**Keywords:** Diets, Nutrition, Markets, India, Cost, Food environment

## Abstract

In this paper, we quantify the divergence in the cost of current diets as compared to EAT Lancet recommendations at the subnational-level in India. We use primary data on food prices and household food purchases, and secondary data on food expenditures for a period of 12 months in 2018–19. The cost of the EAT Lancet dietary recommendations for rural India ranges between $3.00- $5.00 per person per day. In contrast, actual dietary intake at present is valued at around $1.00 per person per day. In order to get to the EAT Lancet recommendations individuals will have to spend nearly $1.00 per person per day more on each of meat fish poultry, dairy foods and fruits. The deficit in current diets relative to recommendations is marked by seasonal variations driven by volatility in the underlying food prices. This paper extends the evidence base for the affordability of the EAT Lancet diet to a subnational-level in India, using the most recent data on prices and expenditures, over time. We highlight the need for tracking rural markets at the subnational level, over time for their nutritional quality and ability to provide affordable, nutritious diets to the poor. Crop diversification, investments in rural infrastructure and well-functioning markets can move rural India towards more nutrition sensitive food environments.

## Highlights

• There is a significant gap between current food consumption and the EAT Lancet diets for India.
• The cost of the EAT Lancet diets is $3.00- $5.00 per person per day in rural India.
• Currently, individuals are consuming the equivalent of $1.00 per person per day.
• Additional spending of $1 each is needed in nutrient- dense foods such as meat/fish/poultry, dairy & fruits.
• Seasonal fluctuations in price and costs are high for Fruits, Green Leafy Vegetables & other vegetables.
• This paper uses primary and secondary data on monthly food consumption and prices at the subnational level for 2018–19.

## 1 Introduction

One in every three persons suffers from diet-related malnutrition, globally. Low quality diets are the number one risk factor driving the global burden of disease (Global Panel on Agriculture and Food Systems for Nutrition, 2016). One of the main constraints to the achievement of nutrition related Sustainable Development Goals globally is the high cost of healthy diets (FAO, 2020). It is estimated that for more than 3 billion people the least-cost healthy diet is out of reach because of high prices of nutritious foods. 1.3 billion of the above 3 billion people are estimated to be living in South Asia (FAO, 2020). In 2019, the EAT- Lancet Commission put forward a set of global recommendations for dietary intake capable of feeding 10 billion people by 2050 while also meeting the objectives of sustainability in health outcomes for both, people and the planet (Willett et al., 2019). More recently, attention has focused on the ability of food systems to provide healthy diets at the least possible cost (FAO 2020). These costs estimates are at the global, regional or national levels using national level averaged annual prices for diets based on either national food-based dietary guidelines or the EAT Lancet guidelines. Such a level of aggregation – spatially and temporally – can mask substantial heterogeneity in the cost of diets across seasons and at a subnational level. Further, the recommended cost of diets estimated for the such least-cost ‘hypothetical diets’ (FAO 2020) have not been compared to actual dietary intake in order to assess how healthy current diets are and how they can meet dietary recommendations like those of the EAT Lancet Commission.

Recently released estimates in the State of Food Security and Nutrition in the World (SOFI) report (FAO 2020) suggest that the EAT lancet diet has a median cost of $3.44 per person per day, globally. It ranges from a minimum of $3.31- $3.61 across four different versions: flexitarian, pescatarian, vegetarian and vegan (FAO 2020). The EAT Lancet reference (Willet et al., 2019) diet is rich in micronutrient-rich foods like fruits, vegetables, whole grains, legumes, nuts and unsaturated oils. It includes moderate levels of seafood and poultry and very little of red meat, refined grains and added sugars. Such a diet amounts to an intake of 2500 kilocalories per person per day^2^ (Willet et al., 2019). In order to meet EAT Lancet recommendations by 2050, the consumption of all food groups except starchy vegetables will need to be increased in South Asia (EAT, 2019).

Price and expenditure data from the World Bank's International Comparison Program (ICP) has been aggresively used for analyzing the affordability of nutritious diets in India. For instance based on ten different national food based dietary guidelines (different from EAT Lancet) a healthy diet ($3.41 per person per day) amounts to 118.2% of per capita food expenditure, making it out of reach for nearly 80% of India's population (FAO, 2020). The usefulness of such estimates is limited in large part due to the data underlying these results. The ICP uses a single, nationally-representative price for each food item (Herforth et al., 2020) that is unlikely to capture actual markets that are available to the rural poor and actual ‘prices paid by the poor’ (Ray, 2015). Furthermore food expenditure data for India in the ICP's 2017 round continues to be sourced from the 2011–12 round of the National Sample Surveys.^3^ The latter data– which is a decade old - has also been used for comparing cost of meeting India's food based dietary guidelines to wage rates (Raghunathan et al., 2020) and by comparing actual calorie intake to EAT Lancet recommendations (Sharma et al., 2020).

Two other aspects that are missing in current estimates relate to seasonality and subnational units as the level of estimation. Although it is well recognized that there can be variations in the availability, prices and purchase of food items over time (FAO 2020), recent estimates of cost of diets based on these indicators are restricted to annual averages (Herforth et al., 2020). Little therefore is known about seasonality in the cost of diets. Thus far estimates of the cost of the EAT Lancet diet have been computed at the global level^4^ (FAO 2020) and disaggregated at the regional level at best (Hirvonen et al., 2020). It has however been shown that there are significant variations in affordability of healthy diets between low, middle and high-income countries (FAO 2020). Similarly it is agreed that food prices and expenditures can vary significantly within a country (Ray 2015; Herforth et al., 2020). This underscores the importance of capturing prices, expenditures and food purchase data at the subnational level.

In this paper, we address these gaps in the literature in the following way. First, we estimate the cost of the EATLancet diet at the district-level in India using high-frequency primary data on food diversity and prices collected monthly from local food markets in our field locations between June 2018–May 2019. Our second objective is to compare the cost of current diets to the cost of EAT Lancet diet from the point of view of ‘ground-truthing’ the affordability of EAT Lancet diets. This allows us to identify what it will take for households to meet dietary recommendations. Little is known about the magnitude of divergence between present diets and EAT Lancet diets in value terms – more so for individual food groups - at the subnational level. We quantify the gap between the two and then tie this gap back to both, price levels of their constituent food groups and seasonal variations in their prices. To our knowledge seasonality in cost estimates for diets (both EAT Lancet and current diets) and the deficit therein has not been investigated for India. And finally, we validate the cost of diet estimates based on primary data against food expenditure data from 2018 to 19 that is representative at the state and national-level. In doing so we move beyond the use of 2011–12 data that has been used for affordability of diets estimates in India (Raghunathan et al., 2020; Sharma et al., 2020; Herforth et al., 2020). By accounting for intra-country heterogeneity in prices, quantities and expenditures from 2018 to 19 over a period of 12 months we are able to assess where diets currently are, relative to recommended intakes and to identify what it will take for households to be able to meet those recommendations.

Section 2 describes the data and methods used in this paper. The results are presented in section 3. Given the extent and nature of the nutrition deficit in diets, in section 4 we make policy recommendations on how food markets and food policy can be made more nutrition-sensitive in order to achieve the EAT Lancet dietary recommendations.

## 2 Methods

### 2.1 Primary data and sites

The primary data for this study was collected as part of the TCI's TARINA program in India over 2018–19. The TARINA program aims to promote nutrition sensitive food systems in four districts of India – Munger (Bihar), Maharajganj (Uttar Pradesh), and Kandhamal and Kalahandi (Odisha).

In 2018- 19 TCI TARINA implemented a market and diet diversity study. The market component of this study carried out a detailed, in-depth assessment of diversity and prices of food items available in 12 local weekly village markets also known as *haats*-across the four TARINA districts (Table 1, Appendix 1). We follow the market survey methodology specified in Pingali and Ricketts (2014). Enumerators visited each market on the market day in the first week of every month and collected data on three aspects: an inventory of all food items that were available in the market, prices of all food items and the number of vendors (by gender) selling each food items. Food availability data was collected for 259 food items belonging to different food groups like cereals, pulses, fruits, vegetables, nuts and seeds, oils and fats, dairy, legumes, spices and meat/fish/poultry (MFP), processed/packaged food items like sweets, beverages and various types of cooked foods. For each food item price data was collected from four randomly selected vendors in each market in every month of the year. Having four price-points for each food item allowed us to capture within-market price differentials that can occur based on factors like quality, seasonality and post-harvest technologies for perishables.

The diets component of the same study surveyed a sample of 160 households (2 villages per district, 20 households per village) every month beginning August 2018–May 2019. These households were a subset of households from each village that were earlier surveyed for a baseline in 2017 and later in 2019. The villages for this component of the market study were selected in relation to proximity to the respective weekly markets. Data was collected on women's dietary diversity, household food purchases (quantity) and women's time use. These households were surveyed following the market day in the survey week. Therefore, the reference period of 7 days was expected to account for the role of foods purchased in the same week that we also collected food price data for.

### 2.2 Secondary data and sites

We use secondary data from Consumer Pyramids Household Survey (CPHS) carried out by the Centre for Monitoring of the Indian Economy (CMIE). The CPHS is a high-frequency, longitudinal survey of household well-being in India. One component of the CPHS is the Consumption Pyramids that specifically tracks consumption expenditure on food and non-food items from a nationally representative sample of 119,000 households across 28 states and union territories of India.^5^ The survey is conducted over a four-month period and monthly expenses are collected for each of the four months that immediately preceded the month of the survey. The food expenditures account for a total of 39 food items that include whole grain cereals, pulses, edible oils, vegetables, fruits, beverages and packaged/processed foods of different types (for details on the inclusions see Table 2, Appendix 1). The Consumption Pyramid also collects information on household size, age and gender of household members.

We use the CPHS data on food expenditures for the period- June 2018 to May 2019 for three states- Uttar Pradesh, Bihar and Odisha. These states represent the ‘lagging’ regions of Eastern India characterized by low agricultural productivity and a high prevalence of malnutrition (P. L. Pingali et al., 2019). These are also the states where we have implemented multiple detailed household surveys over time as described above. All India averages are provided to situate the state-level data relative to the rest of the country. Although it is not directly comparable to the official NSSO surveys on consumption expenditure the CPHS fills a glaring gap in the availability of such statistics, especially given that the last round of NSS data that is publicly available in the country is from 2011 to 12.

### 2.3 Construction of key variables

#### 2.3.1 Cost of the EAT Lancet Dietary recommendations

The cost of reaching the EAT Lancet dietary recommendations is estimated for the period June 2018–May 2019 following the Cost of Recommended Diet (CoRD) methodology (Dizon et al., 2019). This methodology requires data on three aspects of diets: recommended intake of foods, retails prices of foods and factors to adjust the prices by edible portion of each food item. The first of these - the average recommended intake (in grams) of different food groups - was based on the EAT Lancet Dietary guidelines and is summarized in Table 1. We estimate the cost of the EAT Lancet diet for the following nine food groups: starchy staples, pulses, fruits, dark green leafy vegetables, vegetables, MFP, dairy, sweeteners and oils. Including green leafy vegetables, pulses and MFP as distinct food groups permits us to quantify the affordability of specific nutrient-groups at a more granular level as compared to existing estimates that, for example, have combined all fruits and vegetables together as one group (Hirovnen et al., 2019) or merged pulses/legumes and animal-sourced foods together as ‘protein-rich foods’ (FAO 2020). A look at the ICP 2017 data^6^ for India indicates that the price level index is not available for pulses/legumes at all, and is not disaggregated for vegetables.

**Table 1:** Recommended intake of EAT Lancet Commission.

Food group	Recommended intake per person per day in grams	Food group terminology used in this paper
Starchy staples	232	Cereals
Fruits	200	Fruits
Green leafy vegetables	100	Leafy vegetables
Vegetables (excluding GLV)	200	Vegetables
Potatoes and cassava	50	
Legumes	125	Pulses
Beef and Lamb	7	Meat, fish, eggs
Pork	7	
Chicken and other poultry	29	
Eggs	13	
Fish	28	
Dairy	250	Dairy
Fats and oils	51.8	Oils
Sweeteners	31	Sweeteners

The retail prices of food items (four price points per item) were collected monthly from rural markets as described in the previous section. As a first step, an average of the four price points was calculated and treated as the market price for each food item in a given month, market and district. The minimum market price for each food group was based on the identifying the cheapest of its constituent food items. Similarly, the average market price of each food group was based on an average of the market prices of all food items included in that food group. For example, in the month of June in the district of Munger the minimum price for the vegetables food group, based on the cheapest vegetable available, was USD 0.58 per kilogram. At the same time the average price for this food group, based on the prices of all available vegetables in that month, was USD 1.32. These minimum and average prices were first calculated for every market and month combination. They were then averaged across markets to arrive at minimum and average food-group prices for each district-month combination, and for the total sample. All food item prices were converted to price per edible portion by using edible portion factors from the USDA's Food and Nutrient Database for Dietary Studies.^7^ The absence of data on edible portions specifically for use of foods in India is a limitation as it is likely that for some food items the edible portions might not reflect cultural consumption norms. A density factor of 2.0 g/ml for milk powder and 1.0 for yoghurt from FAO's Food Density Database^8^ was used. We also use the same factor for *khoya* which is a version of dried whole milk, locally consumed in these locations. The latter is based on locally used conversion estimates. The use of the edible portions from the USDA and density factors from the FAO is similar to that in Hirvonen et al. (2020) and Raghunathan et al. (2020) respectively.

The CoRD for the EAT Lancet guidelines is calculated as the sum of the cost of achieving the recommended intake for each of its constituent food groups. The latter was calculated as the product of the recommended intake (from Table 1) with food group level prices, per edible portion. We estimate a minimum cost of the EAT Lancet diet that is the sum of the cost of meeting the recommended intake for each food group when minimum food group prices are considered. Similarly, an average cost of the EAT Lancet diet was based on average food group prices. These estimates were generated for every district and every month, as well as the total sample. Prices in Indian Rupees (INR) were converted to US dollars using the World Bank 2019 PPP Conversion factor^9^ of 18.4 for INR to USD. We then compare the cost of EAT Lancet diet to both, the international poverty line of USD 1.90 and India poverty line of USD 1.8.^10^

Some food groups like dairy were completely absent in one or more months across districts. In that case, the price of dairy for that month is imputed as the average of the price of dairy in the preceding and following month. We do this because our market survey accounts for one week of the month, therefore it is possible that these food groups may have been available at other points of time during the same month. In our computation of the cost of the EAT Lancet diet we replace finger millet *(ragi*) and maize with rice and/wheat in some months to reflect foods that are locally preferred and consumed.

Seasonality in the cost estimates for the EAT Lancet recommendations is calculated by comparing the percentage change in cost relative to the month of June 2018. A similar exercise is done to analyze seasonality in food group prices.

#### 2.3.2 Affordability of diets based on household-level primary data from TCI TARINA

**Fig. A2.1:**
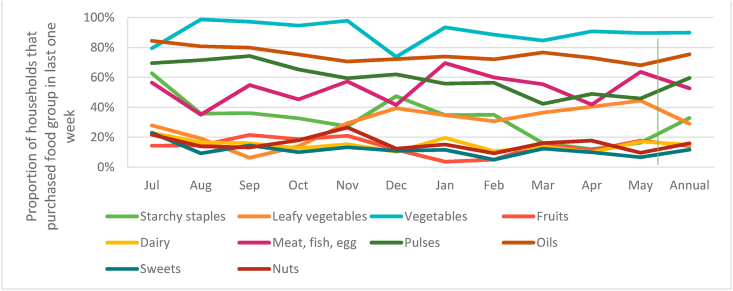
Household reliance on markets for food purchases

**Fig. A2.2:**
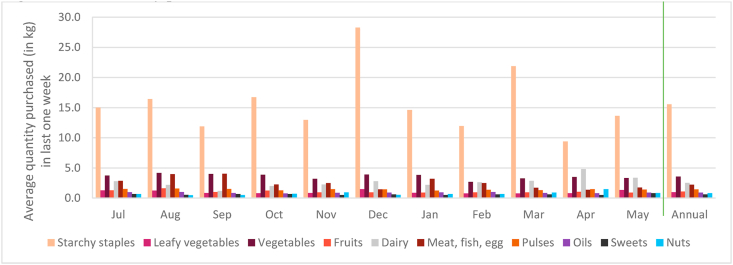
Quantity purchased.

Additionally, for a longitudinal sample of 160 households, we bring together data on quantities purchased (in the last 7 days) and combine that with the price data from weekly markets to estimate weekly total food expenditures. Using household size, we then calculate a per person per day cost of diet for these households. We are able to compute a minimum and average cost of actual diet using the minimum and average food item prices for the ten-month period August 2018–May 2019. The cost estimates are limited to the extent that our per capita estimates do not account for the specific nutrient requirements of different household members that might differ by age, gender and other physiological needs. Detailed data on monthly household reliance on food markets is provided in Fig. A2.1, Fig. A2.2, appendix 2. The cost of diets from this sample of 160 households is validated against food expenditure data from a larger sample of 3600 households that were surveyed by TCI- TARINA in May 2019 (appendix 5).

The cost estimates for current diets are compared to the cost estimates for the EAT Lancet diet and the difference between the two is identified as a deficit. This deficit is computed for the total cost and for individual food groups as well.

#### 2.3.3 Actual expenditure on diets based on nationally representative data

We use nationally representative data in order to validate our results from section 3.3.2 at the population level. The CMIE- CPHS data on food expenditures is collected for the following food groups: cereals, pulses, fruits, vegetables including wet spices, milk and milk products, meat, eggs and fish, potatoes and onions, edible oils and ghee. In order to be able to compare household food expenditures to the cost of the EAT Lancet diet, the data on the former was aligned with the food groups included in the latter. Accordingly, the category on potatoes and onions was combined with that of vegetables, and the categories edible oils and ghee were included together for the food group ‘fats and oils’. We also note that it is not possible to separate out green leafy vegetables (GLV) from vegetables in the CMIE data.

We estimated the per person per day average expenditure for each individual by dividing the monthly household food expenses by the household size and number of days in a month. These estimates are averaged at the state and national level for the months of June 2018–May 2019. The per person per day expenditures were also disaggregated to arrive at the expenditure on each of the constituent food groups. Expenditures in INR are converted to USD in PPP by using the conversion rates of World Bank and OECD databases: 20.986 for 2018 and 21.107 for 2019. These CPHS estimates are compared to actual cost estimates from section 3.3.2 and to the cost of the EAT Lancet diet estimates from section 3.3.1.

## 3. results

3

### 3.1 Descriptive statistics

The average cost of the EAT Lancet diet based on minimum and average prices is $3.33 and $5.32 per person per day. In comparison, the cost of actual diets is $0.62 and $1.00 per person per day. That is lower than the population level expenditure on diets on average across the country ($1.74) and each of our three locations. MFP are the most expensive food group followed by oils and dairy. In contrast the prices of staple cereals are much lower. (Table 2). Readers are referred to Table A3.1, Table A3.2, Table A3.3, Appendix 3 for district-level descriptive statistics.

**Table 2:** Descriptive statistics.

		Minimum	Maximum	Average
Cost of diets (USD per person per day, 2019 PPP)				
EAT Lancet diet *Min*		$2.87	$3.49	$3.33
EAT Lancet diet *Avg*		$5.14	$5.60	$5.32
Actual diets *Min*		$0.46	$0.84	$0.62
Actual diets *Avg*		$0.76	$1.33	$1.00
Population level diets from CPHS				
All- India		$1.68	$1.82	$1.74
Bihar		$1.41	$1.49	$1.45
Uttar Pradesh		$1.27	$1.64	$1.38
Odisha		$1.21	$1.38	$1.30
Food group price (USD, 2019 PPP)				
Cereals	*Min*	0.90	1.38	1.20
	*Avg*	1.50	2.17	1.83
Leafy vegetables	*Min*	0.52	1.29	0.95
	*Avg*	0.88	1.62	1.27
Vegetables	*Min*	0.32	0.71	0.57
	*Avg*	1.16	1.70	1.53
Fruits	*Min*	0.86	2.37	1.60
	*Avg*	2.21	4.93	3.32
Dairy	*Min*	3.34	4.54	3.73
	*Avg*	3.34	4.54	3.95
Meat, Fish, Poultry	*Min*	6.77	9.29	7.41
	*Avg*	10.21	13.15	11.57
Pulses	*Min*	1.81	2.65	2.23
	*Avg*	3.04	3.54	3.31
Oils	*Min*	4.92	5.39	5.21
	*Avg*	9.49	12.41	10.89
Sweeteners	*Min*	1.91	2.13	2.05
	*Avg*	2.12	2.44	2.28
Nuts	*Min*	2.67	5.84	3.94
	*Avg*	10.43	25.96	16.26

Note: Min refers to minimum. Avg refers to Average. All values are averaged for the total sample for the time period June 2018–May 2019.

### 3.2 Cost of the EAT lancet diet

**Fig. 1:**
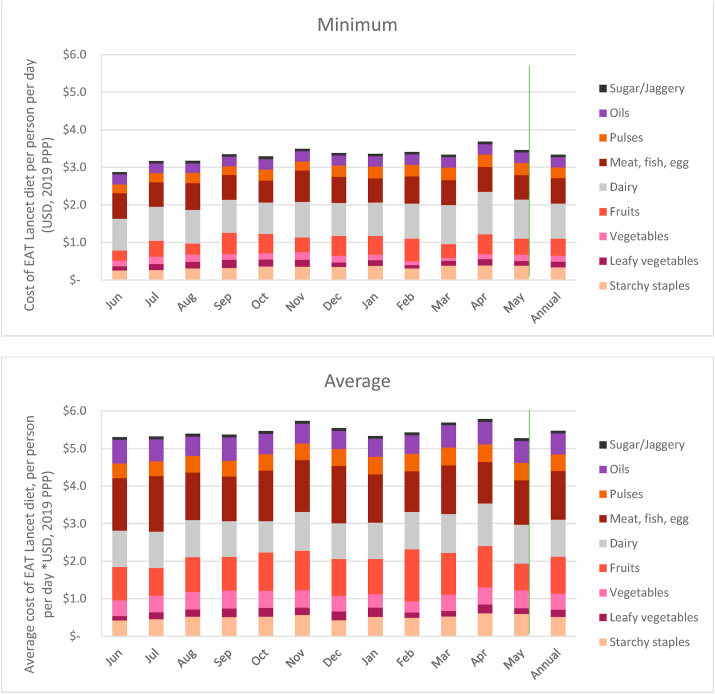
Cost of EAT Lancet diet, June 2018–May 2019. Note: *Minimum cost* refers to cost estimated taking into account the cheapest food items in each food group. *Average cost* refers to cost estimates taking account the average price of all food items in each food group. Prices are adjusted for edible portions. Vertical line demarcates annual average from monthly data.

We find that on average it would have cost an individual at least $3.30 per day to meet the EAT Lancet recommendations if she buys the cheapest food item(s) in each food group from the weekly village market in each district (*yearly* in Fig. 1). The cost of the EAT Lancet diet exceeds $5.00 per day if she chooses to purchase foods that are of average cost within each food group. A list of foods that make up the minimum and average cost estimates is provided in Table A3.4, Table A3.5, appendix 3. Both cost estimates for the EAT Lancet diet are greater than the World Bank ($1.90) and India-specific poverty lines ($1.80). These results are similar to the cost estimate of approximately $3.50 per person per day globally for the EAT Lancet diet (FAO, 2020).

As compared to the minimum costs, the average cost of some food groups like fruits and MFP nearly doubles in each district while that for vegetables goes up by a factor of three. There is considerable spread in the cost estimates as is evident from the standard deviation of the cost of individual food groups (Table A4.1, Table A4.2, Appendix 4). The variation in cost estimates is significantly higher for fruits and MFP compared to all other food groups.

**Fig. A4.1:**
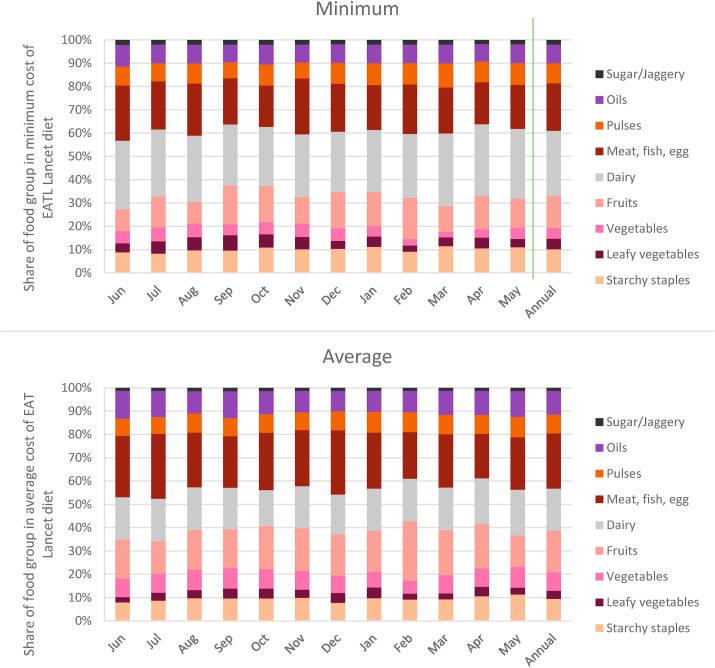
Food group shares in cost of EAT Lancet diet, June 2018–May 2019.

The two food groups that account for the largest share of the minimum cost are meat/fish/egg (nearly 30%) and dairy (20%) (Fig. A4.1, Appendix 4). In the case of dairy this corresponds to its share of 18.8% in total recommended daily intake (grams). However, the cost share for MFP is significantly higher than the share of MFP in total daily recommended intake of 6.3%. A similar contrast can be seen for vegetables that account for 5–8% of the cost of the EAT Lancet diet even though their share in recommended intake is 15%. The cost share of other food groups like GLV, fruits and pulses is in line with the quantities recommended for daily intake.

**Fig. 2:**
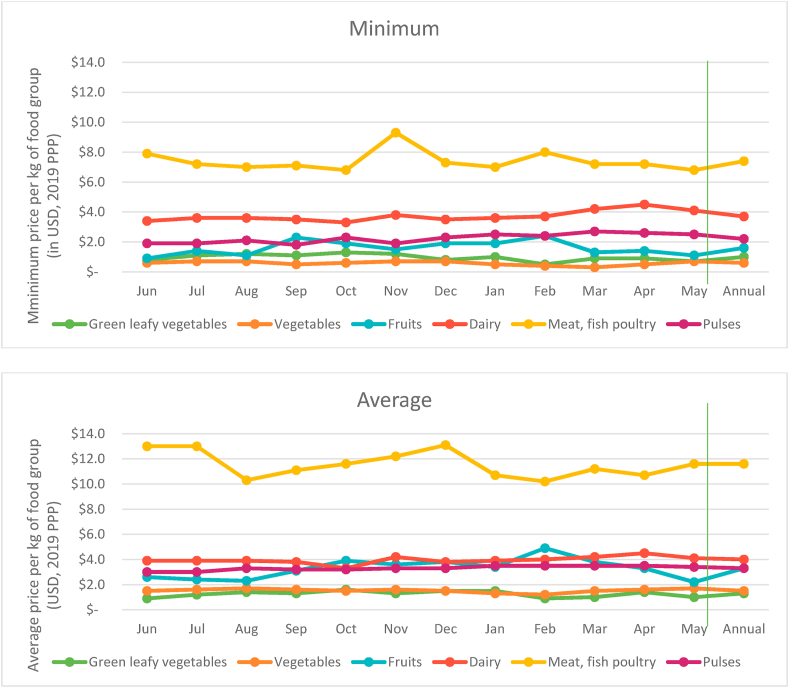
Monthly retail prices of non-staple food groups, June 2018–May 2019. *Note: Minimum* refers price of the cheapest food items in each food group. *Average* refers to the average price of all food items in each food group. Vertical line demarcates annual average from monthly data.

Such differences in cost shares of different food groups, relative to their share of total recommended intake can be understood in terms of the variation in their prices (Fig. 2). MFP are the most expensive, costing nearly $12 per kg on average, over a 12-month period. This is followed by dairy products that hover around $4 per kg in most months. This relatively high price of dairy can be explained by the near absence of milk which is cheaper than dairy items that are usually available like yoghurt/milk powder/*khoya*. Fruits on average cost a little more than $3 per kg, similar to the prices of pulses. Vegetables are one of the cheapest food groups in rural markets, explaining their low cost share relative to share of recommended intake.

### 3.3 Comparison of cost of EAT lancet diet to cost of actual diets using TCI- TARINA data

**Fig. 3:**
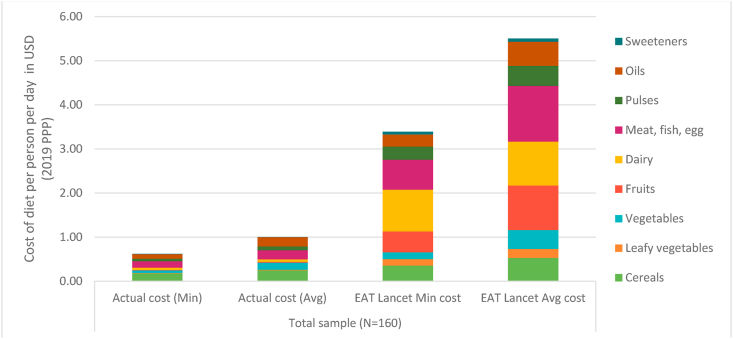
Comparison of cost of actual and recommended diets, August 2018–May 2019. Note: *Min* refers to cost estimated taking into account the cheapest food items in each food group. *Avg* refers to cost estimates taking account the average price of all food items in each food group. Actual cost uses data on quantities purchased multiplied by prices. EAT Lancet cost uses recommended quantities. Prices are adjusted for edible portions.

**Fig. A5.1:**
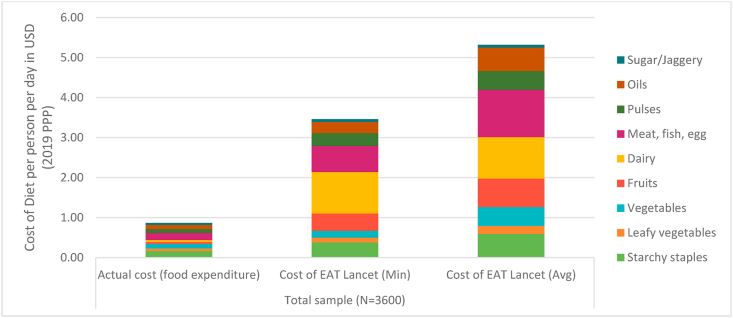
Cost of diet estimates for actual and recommended diets in rural India, May 2019.

In this section, we present results related to actual food expenditures for the 160 TARINA households that were surveyed every month. For these households, the cost of actual individual dietary intake is equivalent to $0.60- $1.00 per person per day, averaged over August 2018–May 2019 (Fig. 3). In order to meet EAT Lancet recommendations households would have to spend $2.80 to $4.30 *more* per person per day. These cost estimates are in line with average expenditures of a larger sample of 3600 households that were surveyed in 2019 (appendix 5, Fig. A5.1). A comparison of actual costs to the minimum estimate for the EAT Lancet diet indicates that individuals are unable to meet even the lower end – in value terms – of the recommended intakes, suggesting that the consumption of all food groups falls short of the average intake recommended by the EAT Lancet diet.

**Fig. 4:**
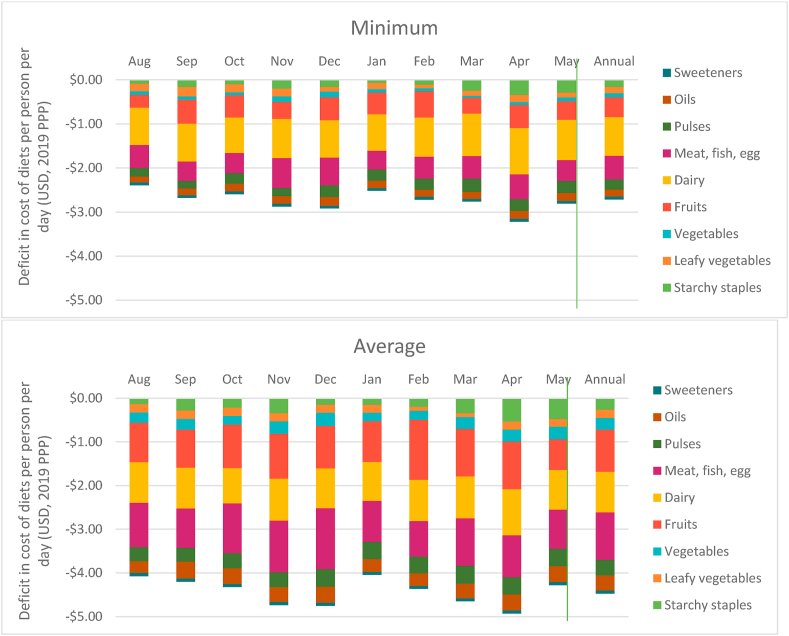
Monthly differences in cost of actual diet vs EAT Lancet diet, August 2018–May 2019. Note: Deficit in cost of diet is calculated as (Cost of actual diet – Cost of EAT Lancet diet). *Minimum deficit* refers to cost estimated taking into account the cheapest food items in each food group. *Average deficit* refers to cost estimates taking account the average price of all food items in each food group. Prices are adjusted for edible portions. Vertical line demarcates annual average from monthly data.

The monthly deficit in cost of actual consumption relative to what is recommended is shown in Fig. 4. Based on minimum price, the gap in diet costs was as much as $2.40 per person per day in August. This increases to nearly $3.00 in November and December and exceeds it by April. Parallel trends can be seen with average prices as well wherein households are spending nearly $5.00 less per person per day (averaged yearly) than the cost of the EAT Lancet diet.

**Fig. 5:**
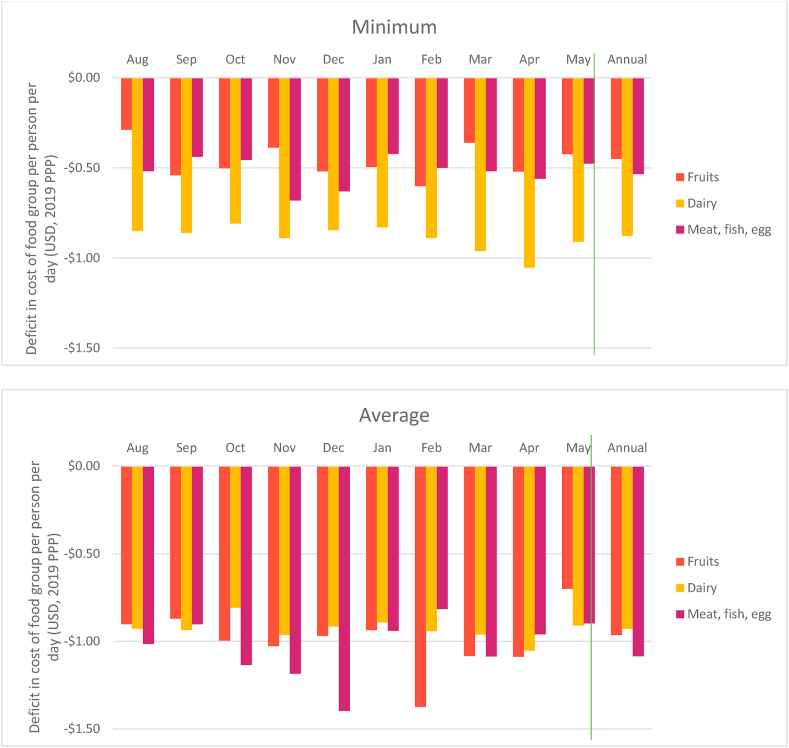
Seasonal trend in difference in cost of non-staples for actual and recommended intake. Note: Deficit in cost of diet is calculated as (Cost of actual food group – Cost of EAT Lancet food group). *Minimum deficit* refers to cost estimated taking into account the cheapest food items in each food group. *Average deficit* refers to cost estimates taking account the average price of all food items in each food group. Prices are adjusted for edible portions. Vertical line demarcates annual average from monthly data.

**Fig. A5.2:**
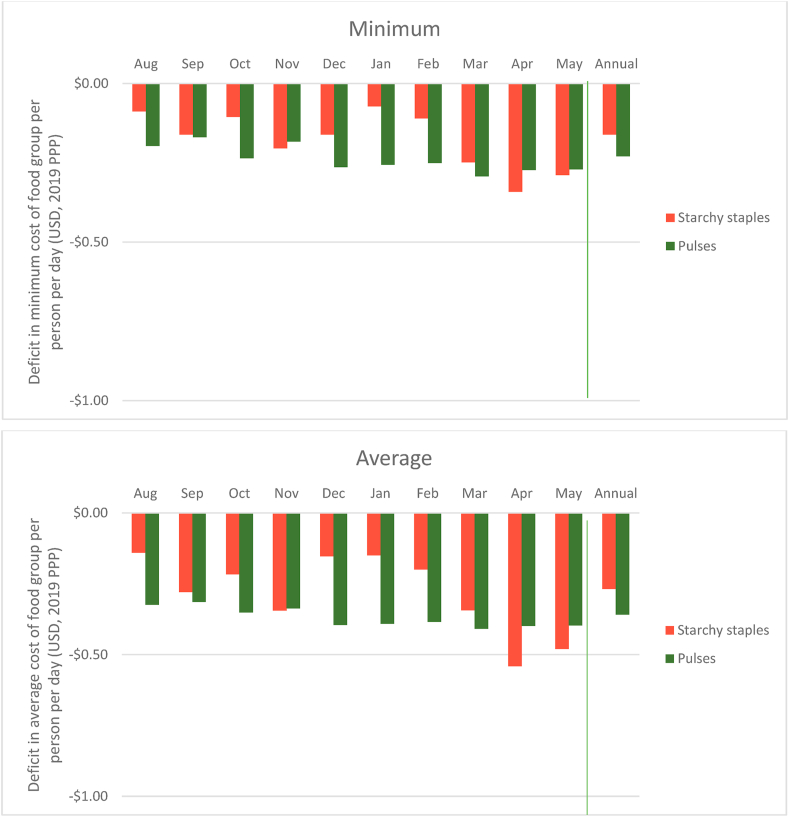
Seasonal trend in actual vs recommended cost of staples.

In order for individuals to meet the EAT Lancet recommendations the greatest increase in spending is required in three non-staple food groups: MFP, fruits and dairy (Fig. 5). (It is also unlikely that this shortfall can be met by own-production adequately). If we look at minimum prices, the deficit in spending is 90 cents per person per day on dairy and 50 cents each for MFP and fruits. When we look at average prices these differences reach $0.96 each for fruits and dairy and exceed $1.00 per person per day for MFP. These results are in line with the estimated increase in expenditure by $1.00 on average on each of dairy, protein-rich foods and fruits that will be required to achieve healthy diets in South Asia (FAO, 2020). The deficit for MFP is highest during October–December and for fruits during February–April periods (Fig. 5). These are also the months when the average price for these food groups peaks. In contrast, the shortfall in expenditure on staples like cereals and pulses is relatively lower (Fig. A5.2 in appendix 5).

### 3.4 Seasonality in cost of diets and food prices

**Fig. 6:**
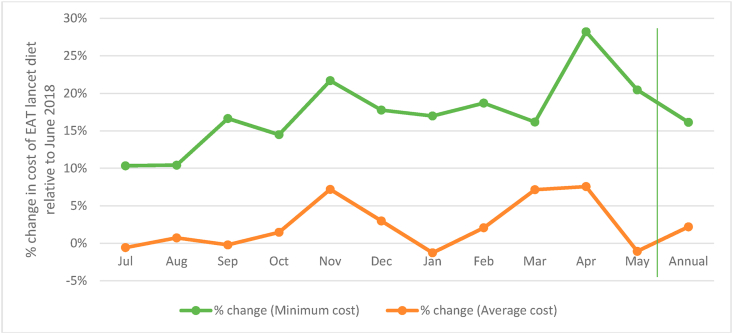
Monthly variation in cost of EAT Lancet diet relative to June 2018. Note: *Minimum cost* refers to cost estimated taking into account the cheapest food items in each food group. *Average cost* refers to cost estimates taking account the average price of all food items in each food group. Prices are adjusted for edible portions. Vertical line demarcates annual average from monthly data.

Seasonality in the cost of the EAT Lancet diet (minimum) is evident as it increases from $2.90 per person per day in June to $3.50 in November and then to $3.70 in April (Fig. 1). This amounts to an increase of 22% and 28% respectively (Fig. 6). While the average cost of the EAT Lancet diet too peaks to nearly $6.00 per person per day in the months of November and April, the seasonal variations based on average prices are much lower in general.

**Fig. 7:**
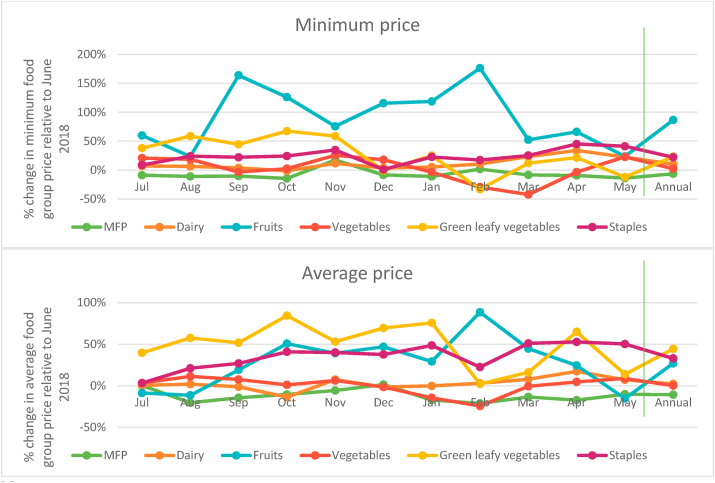
Monthly variation in cost of food groups relative to June 2018. Note: *MFP refers to Meat, Fish and Poultry.* Vertical line demarcates annual average from monthly data.

The fact that the gap between the cost of the recommended and actual diets is also the highest in the same months (Fig. 4 above) when the cost of the EAT Lancet diet peaks indicates that households are spending the least on current diets in these same months, presumably being driven by two factors. The first of these relates to prices of food groups. Fig. 7 shows the variations in minimum and average food group prices for the period July 2018–May 2019 relative to their price in June 2018. While perishables like fruits and vegetables have lower absolute prices (as discussed in section 4.1) they are marked by greater month to month volatility as compared to relatively more expensive non-staples like MFP and dairy products. The months of November and April when the minimum cost of the EAT Lancet diet was 20% more costly (as compared to June 2018) are also the months when prices of food groups are most volatile. In November, the minimum MFP and dairy prices were higher by 18% and 12% respectively. At the same time fruits, GLVs and vegetable prices were higher by 76%, 60% and 26% respectively.

While monthly variations in food group prices can explain variations in the cost of the EAT Lancet diet and the gap relative to current diets, another relevant factor is the stage of the agricultural production cycle. The peaks in variations of average price that occurs in the months of October–November and March–April corresponds to the time just before the beginning of the harvest season. It is likely that both, supplies and household incomes are low at the end of the lean season, contributing to the price volatility and lower purchases respectively.

### 3.5 Validation of cost of current diets to population level estimates based on CPHS data

**Fig. 8:**
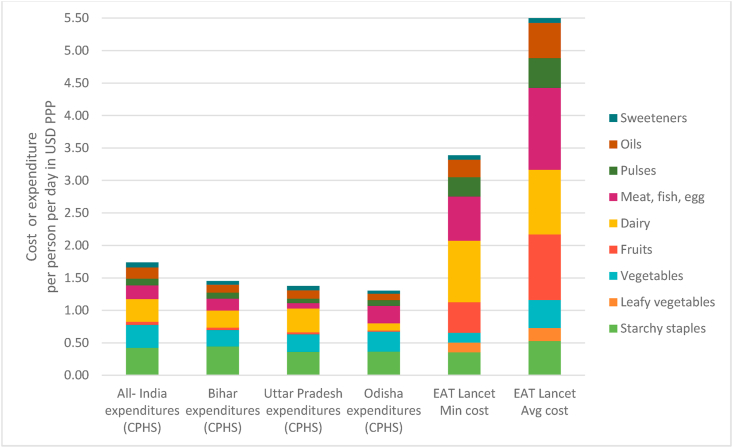
Comparison of CMIE expenditures and EAT Lancet cost estimates (Avg. June 2018–May 2019). *Note:* Green leafy vegetables are merged with vegetables in the CMIE averages for all- India, Bihar, Uttar Pradesh and Odisha. The EAT Lancet cost estimates are based on primary data on rural retail prices of foods in Bihar, Odisha and Uttar Pradesh. These are therefore more comparable to expenditure data for these three states than to the all- India average. CPHS refers to the Consumer Pyramids Household Survey of the CMIE. Min and Avg refer to minimum and average costs respectively.

Food expenditure data for the EAT Lancet food groups from the CPHS indicates that on average rural households in the country were consuming the equivalent of $1.74 per person per day during the period June 2018–May 2019. The cost of diet in all three of our program states was lower than the national average (Fig. 8). Rural households were spending at the very least, half of the minimum cost of EAT Lancet diet ($3.00) and one-fourth of its average cost ($5.00). Such a deficit however is not seen for all the food groups under consideration.

**Fig. A5.3:**
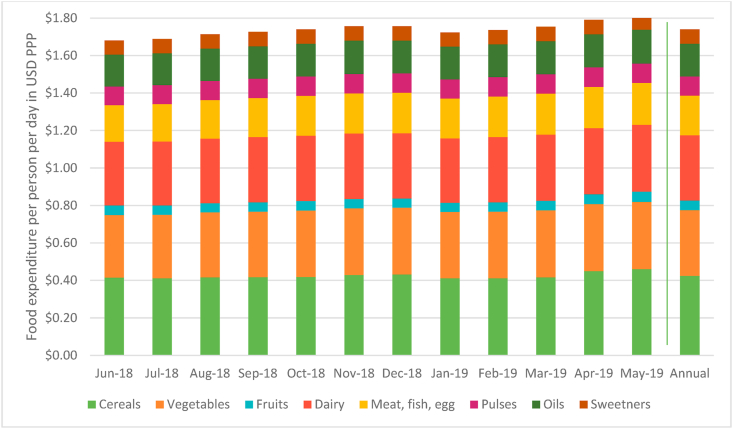
Seasonal variation in All- India rural food expenditures, June 2018–May 2019.

Unlike the results from the monthly TCI- TARINA data, the population level estimates indicate that individuals are able to meet (and even exceed) the recommended cost share for cereals in the minimum EAT Lancet cost estimates. Actual expenditures on vegetables (including GLVs) are also greater than recommended guidelines in Odisha and just shy of the requirement in Bihar and UP. For the remaining food groups, the results from the TCI- TARINA data are reflected at the population as well. People are unable to meet the minimum cost shares of the recommended intake for fruits, dairy and MFP. In UP and Bihar individuals are consuming the equivalent of $0.04 per person per day on fruits as against the recommended $0.47 ($1.01) based on minimum (average) cost. In UP the expenditure on MFP will have to increase by a factor of 8 to achieve recommended intake ($0.08 versus $0.68 per person per day). Similarly, actual expenditure on dairy in Odisha is $0.11 per person per day – nearly one tenth of what is required to meet dietary guidelines for milk and milk products ($0.95). The magnitude of these deficits gets magnified if we compare actual expenditures to average cost of each food group in the EAT Lancet cost estimates. There is little seasonal variation in the cost of actual diet between the period June 2018–May 2019 (Fig. A5.3 in appendix 5). This can, in part, be the result of well-integrated markets that smoothen the seasonal price variations which we would otherwise expect to see.

## 4 Discussion

Food systems in their present form are struggling to provide a healthy diet, especially in low and middle income countries, in the face of stress resulting from factors like population growth, climate change and increasingly scarce natural resources. The interplay of income levels, degree of urbanization and market liberalization, as well as the status of natural resources in countries can aid in prioritizing actions required to reach the goal of healthy diets (Global Panel on Agriculture and Food Systems for Nutrition, 2016). Middle income countries like India need to incentivize a higher intake of fruits and vegetables and ASFs while reducing an excessive intake of calories. The need for such a shift in consumption patterns is also reflected in our results. We show that not only would households need to spend at least three times of what they presently do, in order to get to the EAT Lancet recommendations, but that the bulk of that increase will need to come from non-staples such as MFP, Fruits and Dairy.

Our estimates of the recommended diet based on minimum prices essentially represents the lower-end of the cost required to get to recommended intakes. This is because m*inimum* cost estimates essentially assume that individuals purchase the *cheapest* food item in each food group in order to meet the EAT Lancet recommended intake. When we compare the cost of the recommended diet to the cost of current diets what we find is that the latter is less than the former. In other words, consumption of all food groups falls short of the *average* EAT Lancet recommended intakes. The minimum cost of actual diet in our sample households was $0.60 per person per day. In comparison to recent estimates this is less than the $0.79 per person per day that is required to meet even an energy sufficient diet in India (FAO, 2020). Both, the minimum ($0.60) and average ($1.00) expenditures on dietary intake from our primary sample are also less than the estimated cost of a nutrient-adequate diet ($1.90) for India (FAO 2020).

From a policy perspective, there is a need for convergence of policies in the domains of agriculture, health, education and others in order to improve the affordability of diets in India. Affordability can be ensured by both, increasing incomes as well as making prices of nutritious foods more affordable. On the supply side, a shift towards more nutrition sensitive food systems from the current staple grain fundamentalism policy is the need of the hour. For predominantly agrarian and rural communities, strategies like a diversification of cropping systems can both, ensure diversity of food through own-production as well as through increased incomes that result from crop sales. In India a persistent bias of agricultural policies in favor of staple cereals like rice and wheat has constrained incentives for diversification of the production system (P. Pingali et al., 2017). Diversification of the predominant rice-wheat cropping system in India will depend upon improvements in crop yields, access to inputs like seeds as well as modern technologies and investment in irrigation infrastructure.

Successful diversification also requires the presence of well-functioning markets that reduce price risk and transaction costs for smallholder farmers and ensure adequate price realization. Simultaneous investments in market infrastructure like cold storage facilities that reduce post-harvest food losses of non-staple, perishable food items like fruits, vegetables, dairy and meats are also required. At the same time government procurement of produce needs to take place in a reliable, efficient and remunerative manner. Ensuring well-functioning markets is also critical to ensure that local markets can supply nutritious foods, throughout the year, at affordable prices. Informal and traditional food markets, like the wet markets that are a focus of this paper, need investments (in infrastructure and information) and regulations (eg. related to food safety) to support them in being able to provide perishable foods to low income populations at affordable prices (Global Panel, 2016). Kitchen gardens can also be an important source of diversified and perishable foods like fruits and vegetables throughout the year, thereby reducing seasonal food deficits.

Policy efforts also need to focus on increasing demand for, and consumption of nutritious foods. This can be achieved through behavior change campaigns focusing on nutrition education and empowering women by way of resources and information. These can bring together expertise from health workers, education departments, extension agents amongst others. Our previous work has highlighted the role of women's empowerment in agriculture and nutrition education for improved dietary diversity and reduced iron deficiency in India (Gupta et al., 2019, 2020). A lot of India's poor rely on the country's food safety nets like the Public Distribution System, Integrated Child Development Services and the Mid- Day Meal schemes. These programs can also be used as platforms for providing non-staples for consumption.

Better data systems are required for tracking the role that food markets play in making diverse, nutritious foods available and affordable to the poor. There is increasing evidence that household market integration is a significant determinant of nutritional outcomes such as dietary diversity at the individual and household level (Gupta et al., 2019; Sibhatu and Qaim, 2018). Rural markets can play a significant role in providing diverse diets throughout the year and are less affected by seasonality (P. Pingali and Sunder, 2017). Despite rising evidence of rural market penetration in low income countries, there is very little known about the nature of rural markets and the availability of diverse foods chains (D. Headey et al., 2018; Minten et al., 2014; Reardon et al., 2003). In that respect, our data on 250 food items from rural, village-level *haats* across India sheds light on the ability of local markets to supply nutritious foods throughout the year. In this study, we use detailed primary data on wet markets that serve some of the poorest communities in India. Our market study used a food diversity-and-price that can be replicated to different country/region contexts. Such data collection from local food markets should be high-frequency in nature in order to capture seasonal changes in diversity and volatility of prices of foods. It is agreed that there is considerable variation in prices of foods within countries, especially for perishable, nutrient-rich foods like fruits, vegetables and meats (Herforth et al., 2020). Such food groups are a relatively expensive source of nutrients (Darmon and Drewnowski, 2015; D. D. Headey and Alderman, 2019) and account for nearly 80% of the cost of diets in South Asia (Herforth et al., 2020; Hirvonen et al., 2020).

A recent report by the Global Panel (Global Panel on Agriculture and Food Systems for Nutrition, 2016) shows that South Asia has seen a rapid increase in purchases of processed foods, and that the per capita daily caloric intake of poultry meat and eggs is projected to increase at the rate of 7% and 2.5% per annum between 2005/2006–2030. By bringing in price and expenditure data from 2018 to 19 our results are reflective of such changes in food demand. Information on up to date food prices – more so at the subnational level-can inform not just global food based dietary guidelines like the EAT Lancet but also national ones. It can also inform benchmarks like the international poverty line. A need for adjusting the latter has been highlighted in the recent State of Food Security and Nutrition report given that it falls short of the cost of meeting both, nutrient adequate diets and health diets – globally and for India in particular^11^ (FAO, 2020).

**Fig. A5.4:**
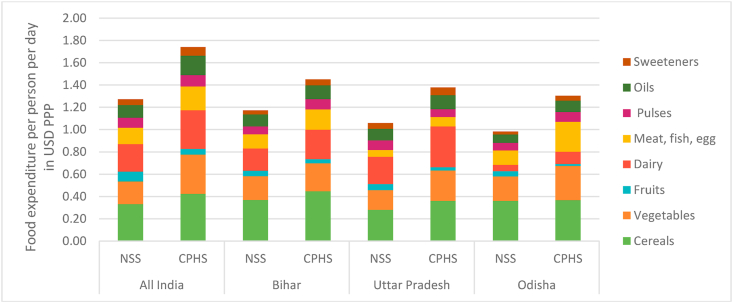
Comparison of food expenditures using NSS (2011–12) and CMIE (2018–19) data.

**Fig. A5.5:**
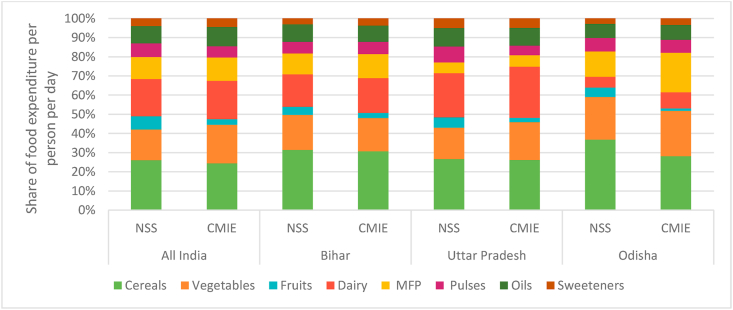
Comparison of share of food expenditures between 2011–12 and 2018–19.

For India one of the biggest data gaps at present relates to the unavailability of recent consumption expenditure data. Officially collected by the NSSO, the publicly available data at present dates back to 2011–12. It is this same food expenditure data that is continues to be used by the ICP with extrapolations having been made using updated PPP rates for 2017. Although not directly comparable, we find that the person per day expenditure on food (EAT Lancet food groups) has increased from $1.20 in 2011–12 (NSS) to $1.75 in 2018–19 (CPHS) on average across the country (Fig. A5.4 in appendix 5). In Bihar, UP and Odisha food expenditure has increased from $1.00 on average to $1.30 over the same period. Households are spending more on every food group except fruits. The share of vegetables, dairy, MFP and oils in food expenditure has increased (Fig. A5.5 in appendix 5). On the other hand, the share of cereals and fruits in total food expenditure has declined while that of pulses is nearly the same in 2018–19 as it was in 2011–12. Such a shift in consumption patterns can be viewed in terms of the Bennett's Law. The food groups that now have a higher share of food expenditure also have a higher income elasticity of demand so it can be presumed that this increase is being driven by economic growth.

Our analysis also highlights the need to account for different market structures that households rely on, at the same time, for their food needs. One of the limitations of our cost estimates is that they assume that the entire diet is being sourced from the same local market. This of course need not always be the case. For example, the absence of milk in some months in our market surveys was primarily explained by the fact that households rely on informal markets for them, like sourcing milk from within their village itself. At the same time the dairy products that are infact available-apart from milk-in *haats* need not be the cheapest. These factors ultimately result in an overestimation of cost of the dairy food group.

And finally, cost of diet estimates can be strengthened by accounting not just for foods purchased from markets but also imputing a cost for food procured from non-market sources. In this regard, our estimates do not account for food from alternative sources like own production, kitchen gardens or even the subsidized staples received through the PDS. While it is likely that the cost estimates would be lower if these sources were accounted for, we believe that the extent of overestimation is not significant. That is because for most perishable non-staples households mainly rely on markets. Related to the distinction between perishables and non-perishables is the frequency of purchase. Households also tend to purchase different food groups with varying frequencies. For instance, cereals are more likely to be purchased in bulk, once or twice a year as opposed to perishables that are likely to be purchased every week or month. This can also explain, in part, why households face lower prices for these food groups as compared to non-cereals. The bulk purchases are reflected in the high average quantities of staples purchased in our sample (appendix 2). One way to account for food from different sources is to design household surveys that ask about food consumption from markets vs non-market sources, at the individual level. In that respect, our estimates of per person per day expenditures are limited in that food may not be distributed equally amongst household members and might not be in line with differing physiological needs that might be better captured through an adult-equivalent approach.

The EAT Lancet Commission highlights the need for a Great Food Transformation in order to reach its recommended targets for healthy diets and the environments. Getting there will require that healthy diets be affordable for people. This paper turns the lens on the ability of food markets in rural India to provide diverse, nutritious and affordable foods to the rural poor. Extending this body of work to different geographies and time periods can provide insight into current diet patterns and relevant future predictions to support the design of evidence-based dietary guidelines and policies to meet those guidelines.

## Funding

This work was supported by the 10.13039/100000865Bill & Melinda Gates Foundation, Seattle, WA [# OPP1137807]. The funding agency was not involved in the study design; collection, analysis and interpretation of the data; in the writing and preparation of the report; and in the decision to submit the article for publication.

## Declaration of competing interest

The authors declare that they have no known competing financial interests or personal relationships that could have appeared to influence the work reported in this paper.

## Figures and Tables

**Table 1:** Market locations for primary data

STATE	DISTRICT		
BIHAR	MUNGER	BANGALWA & DASARTHPUR	
UTTAR PRADESH	MAHARAJGANJ	BANGLA CHARUHA, DHODGHAT CHAURUHA, LEHRA & SATNAGAR	
ODDISHA	KALAHANDI,	G. UDAYAGIRI & TIKABALI	
	KANDHAMAL	BUDDHADHARA, DUNDELMAAL, SAGDA & TENTELKHUNTI	

**Table 2:** List of food items included in the CPHS

S. No.	Categories	Food Groups Included
1.	Cereals	Wheat, Rice, Jowar, Bajra, Ragi, Maize, Barley and other similar grains, Atta, Maida, Besan, Sattu, Bhel, Poha, Mumra, Sabudana, Soya Flour
2.	Pulses	Arhar, grams of all kinds i.e. all kinds of channa, urad, moong, masoor, moth, beans like rajma, etc.
3.	Oils	Edible Oils (Dalda, Soyabean oil, Groundnut oil, Mustard oil, Coconut oil, Olive oil, Palmolein oil, etc), Ghee
4.	Vegetables	Green leafy vegetables, cabbages, cauliflower, lemons, tomatoes, all varieties of pumpkins, roots such as beetroot, carrots, radish, tapioca, etc., legumes such as fresh peas/beans, sprouts, etc., all common vegetables such as brinjal, drum sticks, okra or bhindi, capsicums, etc. It also includes fresh spices such as coriander or dhania leaves, curry leaves, fresh chillies, fresh ginger, fresh pepper, fresh turmeric, lime or nimbu, potatoes and Onions
5.	Fruits	Bananas, pineapples, guava, man- goes, grapes, melons, papayas, apples, pears, lichis, cherries, peaches, and citrus fruits such as oranges, sweet lime, strawberries, other berries, kiwi fruit, passion fruit, fresh dates, etc. Fresh fruits also include cut fruits. It also includes expenses on canned fruits.)
6.	Milk & Milk Products	Loose milk, packed milk such as in polythene bags, tetra-packed milk or any other packaging of milk, powdered milk like Amul or Nestle milk powder; Milk products include expenses on the purchase of curds, butter milk, lassi, cream, cheese, paneer and other dairy products.
7.	Meat, Eggs and Fish	Chicken, mutton, pork, beef and other animal meats, and all kinds of fish including fresh water and sea fish. This includes dried or canned meat and fish as well. This includes purchase of the whole or cut pieces of meat or fish. It does not include the purchase of cooked meat preparations. Purchase of frozen semi-cooked meat/fish or processed meat/fish packs are also not included here. These are included under ready to eat foods.
8.	Sweeteners	Sweeteners (Sugar, gur/jaggery, khandsari, honey and sugar-free or zero-calorie sweeteners that are available in the form of tablets or in sachets.

Note: The CPHS also collects information on processed, purchased foods not included in this analysis.

**Table A3.1:** Cost of EAT Lancet diet (Per Person per day, USD PPP, 2019)

District	Cost of EAT Lancet diet	Cereals	Leafy vegetables	Vegetables	Fruits	Dairy	Meat, fish egg	Pulses	Oils	Sweeteners	Total
Munger	*Min*	$0.3	$0.1	$0.1	$0.4	$0.8	$0.7	$0.2	$0.3	$0.1	$3.1
	*Avg*	$0.5	$0.2	$0.4	$1.0	$1.0	$0.9	$0.4	$0.6	$0.1	$4.9
Maharajganj	*Min*	$0.3	$0.1	$0.1	$0.3	$1.0	$0.6	$0.4	$0.3	$0.1	$3.2
	*Avg*	$0.7	$0.1	$0.4	$0.8	$1.0	$1.7	$0.5	$0.7	$0.1	$5.9
Kandhamal	*Min*	$0.4	$0.2	$0.2	$0.5	$1.0	$0.9	$0.3	$0.3	$0.1	$3.7
	*Avg*	$0.4	$0.2	$0.5	$1.0	$1.0	$1.4	$0.5	$0.6	$0.1	$5.6
Kalahandi	*Min*	$0.3	$0.2	$0.2	$0.5	$1.0	$0.6	$0.3	$0.3	$0.1	$3.5
	*Avg*	$0.4	$0.3	$0.5	$1.2	$1.0	$1.2	$0.5	$0.4	$0.1	$5.6
Overall	*Min*	$0.3	$0.2	$0.2	$0.5	$0.9	$0.7	$0.3	$0.3	$0.1	$3.3
	*Avg*	$0.5	$0.2	$0.4	$1.0	$1.0	$1.3	$0.4	$0.6	$0.1	$5.5

Note: *Min* refers to cost estimated taking into account the cheapest food items in each food group. *Avg* refers to cost estimates taking account the average price of all food items in each food group. Prices are adjusted for edible portions.

**Table A3.2:** Cost of actual diets (Per Person per day, USD PPP, 2019)

District	Cost of actual diet	Cereals	Leafy vegetables	Vegetables	Fruits	Dairy	Meat, fish egg	Pulses	Oils	Sweeteners	Total
Munger	*min*	$0.29	$0.00	$0.09	$0.01	$0.12	$0.11	$0.04	$0.07	$0.00	$0.74
	*avg*	$0.44	$0.01	$0.20	$0.03	$0.14	$0.13	$0.07	$0.17	$0.00	$1.19
Maharajganj	*min*	$0.04	$0.01	$0.05	$0.00	$0.04	$0.05	$0.07	$0.10	$0.01	$0.37
	*avg*	$0.08	$0.01	$0.15	$0.01	$0.04	$0.11	$0.09	$0.27	$0.01	$0.78
Kandhamal	*min*	$0.21	$0.00	$0.08	$0.00	$0.02	$0.31	$0.05	$0.10	$0.00	$0.77
	*avg*	$0.23	$0.01	$0.19	$0.00	$0.02	$0.40	$0.08	$0.20	$0.00	$1.13
Kalahandi	*min*	$0.17	$0.01	$0.04	$0.01	$0.01	$0.12	$0.06	$0.14	$0.00	$0.57
	*avg*	$0.25	$0.02	$0.12	$0.02	$0.01	$0.20	$0.09	$0.19	$0.00	$0.91
Overall	*min*	$0.18	$0.01	$0.06	$0.01	$0.05	$0.15	$0.06	$0.10	$0.00	$0.62
	*avg*	$0.25	$0.01	$0.16	$0.02	$0.06	$0.21	$0.09	$0.21	$0.00	$1.00

Note: *Min* refers to cost estimated taking into account the price of the cheapest food items in each food group. *Avg* refers to cost estimates taking account the average price of all food items in each food group. Prices are adjusted for edible portions.

**Table A3.3:** Food group prices per kg, in edible-portion terms (USD, PPP, 2019)

Food group		Munger	Maharajganj	Kandhamal	Kalahandi	Overall
Cereals	*min*	$1.1	$1.2	$1.4	$1.2	$1.2
	*avg*	$1.7	$2.5	$1.6	$1.6	$1.8
Green Leafy vegetables	*min*	$0.9	$0.6	$1.2	$1.1	$1.0
	*avg*	$1.1	$0.9	$1.4	$1.7	$1.3
Vegetables	*min*	$0.5	$0.4	$0.7	$0.6	$0.6
	*avg*	$1.3	$1.4	$1.7	$1.8	$1.5
Fruits	*min*	$1.5	$1.0	$1.8	$2.1	$1.6
	*avg*	$3.0	$2.5	$3.6	$4.3	$3.3
Dairy	*min*	$3.3	$3.9	$3.9	$4.1	$3.7
	*avg*	$4.0	$3.9	$3.9	$4.1	$4.0
Meat, fish, egg	*min*	$6.4	$7.4	$10.1	$5.8	$7.4
	*avg*	$7.6	$15.6	$13.4	$9.6	$11.6
Pulses	*min*	$1.7	$2.8	$2.2	$2.2	$2.2
	*avg*	$2.8	$3.7	$3.4	$3.4	$3.3
Oils	*min*	$5.0	$5.0	$5.4	$5.5	$5.2
	*avg*	$10.8	$13.3	$11.3	$8.2	$10.9
Sweeteners	*min*	$1.9	$1.9	$2.2	$2.1	$2.0
	*avg*	$2.0	$2.0	$2.3	$2.8	$2.3

Note: *Min* refers to the price of the cheapest food items in each food group. *Avg* refers to the average price of all food items in each food group. Prices are adjusted for edible portions.

**Table A3.4:** Minimum priced items in each food group

MUNGER	Cereals	Leafy vegetables	Vegetables	Fruits	Dairy	Meat, fish egg	Pulses	Oils	Sweeteners
January	Wheat	Spinach	Cabbage	Orange	Pressed Cottage Cheese	Fish	Lentil	Sunflower Oil	Jaggery
February	Wheat	Spinach	Tomato	Grapes	Pressed Cottage Cheese	Fish	Lentil	Soyabean Oil	Jaggery
March	Wheat	Spinach	Cabbage	Orange	Milk Powder	Fish	Lathyrus	Soyabean Oil	Jaggery
April	Wheat	Spinach	Potato	Grapes	Milk Powder	Fish	Lathyrus	Soyabean Oil	Jaggery
May	Wheat Flour	Amaranthus	Onion	Wood Apple	Milk Powder	Fish	Horse gram	Soyabean Oil	Jaggery
June	Wheat	Amaranthus	Bottle Gourd	Water Melon	Cottage Cheese	Fish	Lathyrus	Rice Bran Oil	Jaggery
July	Wheat	Amaranthus	Bottle Gourd	Blackberry	Butter Milk	Fish	Lathyrus - split	Soyabean Oil	Jaggery
August	Wheat	Amaranthus	Bottle Gourd	Banana	Pressed Cottage Cheese	Fish	Lathyrus	Soyabean Oil	Jaggery
September	Wheat	Amaranthus	Bottle Gourd	Guava	Pressed Cottage Cheese	Fish	Kidney bean	Soyabean Oil	Jaggery
October	Wheat	Malabar Spinach	Sponge Gourd	Banana	Pressed Cottage Cheese	Fish	Lathyrus	Soyabean Oil	Sugar
November	Wheat	Amaranthus	Lady Finger	Banana	Pressed Cottage Cheese	Fish	Lathyrus	Soyabean Oil	Sugar
December	Wheat	Amaranthus	Potato	Orange	Pressed Cottage Cheese	Fish	Piegon pea	Soyabean Oil	Jaggery
MAHARAJGANJ	Cereals	Leafy vegetables	Vegetables	Fruits	Dairy	Meat, fish egg	Pulses	Oils	Sweeteners
January	Wheat	Lamb's Quarters	Bottle Gourd	Banana	Milk Powder	Fish	Piegon pea	Soyabean Oil	Jaggery
February	Wheat	Spinach	Cabbage	Banana	Milk Powder	Fish	Peas - split	Mustard Oil	Jaggery
March	Wheat	Lamb's Quarters	Cabbage	Banana	Milk Powder	Fish	Chickpea	Mustard Oil	Jaggery
April	Refined Flour	Other Leafy Vegetables	Cabbage	Banana	Milk Powder	Fish	Chickpea	Mustard Oil	Jaggery
May	Refined Flour	Amaranthus	Cabbage	Water Melon	Milk Powder	Fish	Chickpea	Mustard Oil	Jaggery
June	Wheat	Amaranthus	Onion	Water Melon	Milk Powder	Fish	Lentil	Soyabean Oil	Sugar
July	Wheat	Water Spinach	Cucumber	Guava	*	Fish	Peas - split	Soyabean Oil	Sugar
August	Wheat	Amaranthus	Cassava	Guava	*	Fish	Lentil	Soyabean Oil	Jaggery
September	Refined Flour	Amaranthus	Bottle Gourd	Banana	Milk Powder	Fish	Chickpea	Soyabean Oil	Sugar
October	Refined Flour	Amaranthus	Ivy Gourd	Banana	Milk Powder	Fish	Chickpea	Soyabean Oil	Sugar
November	Refined Flour	Amaranthus	Bottle Gourd	Banana	Milk Powder	Fish	Chickpea	Soyabean Oil	Sugar
December	Refined Flour	Spinach	Green Onion	Banana	Milk Powder	Fish	Chickpea	Mustard Oil	Jaggery
KANDHAMAL	Cereals	Leafy vegetables	Vegetables	Fruits	Dairy	Meat, fish egg	Pulses	Oils	Sweeteners
January	Wheat Flour	Mustard Leaf	Cabbage	Plum	Milk Powder	Fish	Horse gram	Sunflower Oil	Jaggery
February	Refined Flour	Malabar Spinach	Tomato	Orange	Milk Powder	Fish	Horse gram	Sunflower Oil	Sugar
March	Refined Flour	Other Leafy Vegetables	Cabbage	Plum	Milk Powder	Fish	Horse gram	Sunflower Oil	Sugar
April	Refined Flour	Other Leafy Vegetables	Potato	Orange	Milk Powder	Fish	Horse gram	Sunflower Oil	Sugar
May	Refined Flour	*	Onion	Banana	Milk Powder	Fish	Horse gram	Sunflower Oil	Sugar
June	Rice-Unboiled	Other Leafy Vegetables	Cucumber	*	Milk Powder	Fish	Horse gram	Sunflower Oil	Sugar
July	Rice-Boiled	Other Leafy Vegetables	Onion	Mango	Milk Powder	Fish	Horse gram	Sunflower Oil	Sugar
August	Rice-Boiled	*	Onion	Guava	Milk Powder	Fish	Lathyrus - split	Sunflower Oil	Sugar
September	Rice-Boiled	Other Leafy Vegetables	Pumpkin	Guava	Milk Powder	Pork	Horse gram	Clarified Butter/Ghee	Sugar
October	Rice-Unboiled	Malabar Spinach	Raw Papaya	Plum	Milk Powder	Fish	Horse gram	Sunflower Oil	Sugar
November	Refined Flour	Other Leafy Vegetables	Raw Papaya	Plum	Milk Powder	Dry Fish	Horse gram	Sunflower Oil	Jaggery
December	Refined Flour	Malabar Spinach	Brinjal	Muasambi	Milk Powder	Fish	Horse gram	Sunflower Oil	Sugar
KALAHANDI	Cereals	Leafy vegetables	Vegetables	Fruits	Dairy	Meat, fish egg	Pulses	Oils	Sweeteners
January	Refined Flour	Mustard Leaf	Potato	Orange	*	Egg	Horse gram	Sunflower Oil	Sugar
February	Rice-Boiled	Amaranthus	Potato	Plum	*	Egg	Lathyrus	Soyabean Oil	Sugar
March	Wheat Flour	Amaranthus	Tomato	Water Melon	*	Egg	Horse gram	Sunflower Oil	Sugar
April	Rice-Boiled	Amaranthus	Green Onion	Water Melon	*	Egg	Chickpea	Soyabean Oil	Sugar
May	Rice-Boiled	Amaranthus	Green Onion	Raw Mango	*	Egg	Horse gram	Soyabean Oil	Sugar
June	Rice-Boiled	Amaranthus	Onion	Blackberry		Fish	Chickpea - split	Mustard Oil	Sugar
July	Rice-Boiled	Amaranthus	Pumpkin	Mango	Milk Powder	Egg	Lathyrus	Soyabean Oil	Sugar
August	Rice-Boiled	Other Leafy Vegetables	Bottle Gourd	*	Milk Powder	Egg	Horse gram	Soyabean Oil	Sugar
September	Rice-Boiled	Other Leafy Vegetables	Pumpkin	Apple	Milk Powder	Egg	Lathyrus	Rice Bran Oil	Sugar
October	Rice-Boiled	Amaranthus	Pumpkin	Orange	*	Egg	Horse gram	Soyabean Oil	Sugar
November	Rice-Boiled	Other Leafy Vegetables	Pumpkin	Custard Apple	*	Fish	Lathyrus	Sunflower Oil	Sugar
December	Rice-Boiled	Amaranthus	Raw Papaya	Guava	Milk Powder	Egg	Lathyrus	Sunflower Oil	Sugar

**Table A3.5:** Average priced food items in each food group

MUNGER	Cereals	Leafy vegetables	Vegetables	Fruits	Dairy	Meat, fish egg	Pulses	Oils	Sweeteners
January	Rice-Boiled	Amaranthus	Sponge Gourd	Litchi	Milk Powder	Fish	Piegon pea	Sesame Oil	Jaggery
February	Refined Flour	Spinach	Carrot	Pomegranate	Milk Powder	Fish	Black gram - split	Coconut Oil	Jaggery
March	Refined Flour	Spinach	Peas	Apple	Milk Powder	Fish	Black gram	Mustard Oil	Jaggery
April	Rice-Unboiled	Spinach	Beetroot	Apple	Milk Powder	Fish	Lentil - split	Mustard Oil	Jaggery
May	Rice-Unboiled	Amaranthus	Tomato	Jackfruit	Milk Powder	Fish	Piegon pea	Soyabean Oil	Jaggery
June	Rice-Boiled	Amaranthus	Cucumber	Jackfruit	Milk Powder	Fish	Lentil - split	Coconut Oil	Jaggery
July	Rice-Boiled	Amaranthus	Peas	Mango	Milk Powder	Fish	Horse gram	Mustard Oil	Jaggery
August	Rice-Unboiled	Amaranthus	Brinjal	Pineapple	Pressed Cottage Cheese	Fish	Black gram	Mustard Oil	Jaggery
September	Rice-Boiled	Amaranthus	Teasal Gourd	Apple	Pressed Cottage Cheese	Fish	Peas - split	Mustard Oil	Jaggery
October	Rice-Unboiled	Other Leafy Vegetables	Tomato	Apple	Pressed Cottage Cheese	Fish	Horse gram - split	Mustard Oil	Sugar
November	Rice-Boiled	Leafy vegetables	Cucumber	Pineapple	Pressed Cottage Cheese	Fish	Black gram	Mustard Oil	Sugar
December	Refined Flour	Amaranthus	Beetroot	Apple	Milk Powder	Chicken	Lentil - split	Coconut Oil	Jaggery
MAHARAJGANJ	Cereals	Leafy vegetables	Vegetables	Fruits	Dairy	Meat, fish egg	Pulses	Oils	Sweeteners
January	Refined Flour	Spinach	Tomato	Apple	Milk Powder	Chicken	Piegon pea - split	Sesame Oil	Jaggery
February	Rice-Unboiled	Spinach	Cluster bean	Apple	Milk Powder	Chicken	Soyabean	Groundnut/Peanut Oil	Jaggery
March	Refined Flour	Other Leafy Vegetables	Peas	Pomegranate	Milk Powder	Chicken	Black gram - split	Coconut Oil	Jaggery
April	Refined Flour	Spinach	Cluster bean	Water Melon	Milk Powder	Chicken	Soyabean	Coconut Oil	Jaggery
May	Refined Flour	Spinach	Beetroot	Grapes	Milk Powder	Chicken	Black gram - split	Coconut Oil	Jaggery
June	Rice-Unboiled	Spinach	Pointed Gourd	Jackfruit	Milk Powder	Chevon/Mutton	Soyabean	Coconut Oil	Sugar
July	Rice-Unboiled	Water Spinach	French bean	Jackfruit	*	Chevon/Mutton	Black gram - split and dehusked	Sesame Oil	Sugar
August	Refined Flour	Amaranthus	Colocasia	Pear	*	Chicken	Black gram - split	Sesame Oil	Jaggery
September	Rice-Unboiled	Amaranthus	Yam	Jackfruit	Milk Powder	Chicken	Soyabean	Coconut Oil	Sugar
October	Wheat Flour	Amaranthus	Yam	Papaya	Milk Powder	Chicken	Piegon pea - split	Rice Bran Oil	Sugar
November	Refined Flour	Amaranthus	Pumpkin	Apple	Milk Powder	Chicken	Soyabean	Groundnut/Peanut Oil	Sugar
December	Refined Flour	Lamb's Quarters	Cauliflower	Muasambi	Milk Powder	Chicken	Black gram - split	Groundnut/Peanut Oil	Jaggery
KANDHAMAL	Cereals	Leafy vegetables	Vegetables	Fruits	Dairy	Meat, fish egg	Pulses	Oils	Sweeteners
January	Wheat Flour	Mustard Leaf	French bean	Orange	Milk Powder	Dry Fish	Soyabean	Sesame Oil	Jaggery
February	Refined Flour	Mustard Leaf	Peas	Apple	Milk Powder	Fish	Green gram - split	Mustard Oil	Sugar
March	Refined Flour	Other Leafy Vegetables	Lady Finger	Grapes	Milk Powder	Dry Fish	Piegon pea - split	Coconut Oil	Sugar
April	Refined Flour	Other Leafy Vegetables	Lady Finger	Water Melon	Milk Powder	Fish	Piegon pea - split	Coconut Oil	Sugar
May	Refined Flour	*	Pointed Gourd	Jackfruit	Milk Powder	Fish	Green gram - split	Coconut Oil	Sugar
June	Wheat Flour	Other Leafy Vegetables	Peas	*	Milk Powder	Fish	Piegon pea - split	Coconut Oil	Sugar
July	Refined Flour	Other Leafy Vegetables	Raw Papaya	Mango	Milk Powder	Dry Fish	Piegon pea - split	Coconut Oil	Sugar
August	Rice-Unboiled	*	Ivy Gourd	Guava	Milk Powder	Chicken	Green gram - split	Coconut Oil	Sugar
September	Refined Flour	Other Leafy Vegetables	French bean	Guava	Milk Powder	Fish	Piegon pea - split	Mustard Oil	Sugar
October	Rice-Boiled	Malabar Spinach	Ridge Gourd	Grapes	Milk Powder	Pork	Soyabean	Mustard Oil	Sugar
November	Refined Flour	Other Leafy Vegetables	Yam	Grapes	Milk Powder	Fish	Piegon pea - split	Coconut Oil	Sugar
December	Refined Flour	Malabar Spinach	Lady Finger	Grapes	Milk Powder	Dry Fish	Soyabean	Coconut Oil	Sugar
KALAHANDI	Cereals	Leafy vegetables	Vegetables	Fruits	Dairy	Meat, fish egg	Pulses	Oils	Sweeteners
January	Refined Flour	Other Leafy Vegetables	Lady Finger	Apple	*	Chicken	Green gram - split and dehusked	Castor Oil	Sugar
February	Wheat Flour	Mustard Leaf	Raw Papaya	Grapes	*	Fish	Black gram - split and dehusked	Mustard Oil	Sugar
March	Wheat Flour	Amaranthus	French bean	Grapes	*	Dry Fish	Green gram - split and dehusked	Castor Oil	Sugar
April	Wheat Flour	Amaranthus	Raw Banana	Apple	*	Dry Fish	Green gram - split and dehusked	Mustard Oil	Sugar
May	Wheat Flour	Amaranthus	Ivy Gourd	Raw Mango	*	Fish	Chickpea - kabuli	Mustard Oil	Sugar
June	Wheat Flour	Amaranthus	Green Peas	Mango	*	Dry Fish	Black gram - split and dehusked	Coconut Oil	Sugar
July	Wheat Flour	Amaranthus	Ridge Gourd	Orange	Milk Powder	Dry Fish	Piegon pea - split	Mustard Oil	Sugar
August	Refined Flour	Other Leafy Vegetables	Raw Banana	*	Milk Powder	Dry Fish	Piegon pea - split	Sunflower Oil	Sugar
September	Wheat Flour	Other Leafy Vegetables		Grapes	Milk Powder	Dry Fish	Green gram - split and dehusked	Castor Oil	Sugar
October	Refined Flour	Amaranthus	Ridge Gourd	Grapes	*	Chicken	Chickpea - kabuli	Castor Oil	Sugar
November	Wheat Flour	Other Leafy Vegetables	Peas	Grapes	*	Chicken	Green gram - split and dehusked	Castor Oil	Sugar
December	Wheat Flour	Mustard Leaf	Raw Banana	Apple	Milk Powder	Dry Fish	Green gram	Castor Oil	Sugar

**Table A4.1:** Standard deviation based on minimum prices per edible portion (yearly)

	Munger	Maharajganj	Kandhamal	Kalahandi	Total
Starchy staples	0.6740558	1.363056	1.106849	1.674818	1.384218
Leafy vegetables	0.8173143	0.4277039	1.75976	2.112767	1.625395
Vegetables	0.8955801	0.8311687	1.022738	0.7929327	0.9946486
Fruits	2.957859	1.476973	3.275723	5.774308	3.852595
Dairy	3.242769	0.5544593	3.013018	0.66061	2.760117
Meat, fish, egg	1.766164	1.341455	3.29372	1.174878	2.855192
Pulses	1.093684	0.6979474	0.5515113	1.530903	1.370274
Oils	0.1955407	0.2640663	0.3462712	0.3596895	0.3570834
Sugar/Jaggery	0.1028262	0.0732832	0.0346678	0.0257884	0.0952484
Total	4.871839	4.974426	5.766265	4.450862	5.067476

**Table A4.2:** Standard deviation based on average prices per edible portion

	Munger	Maharajganj	Kandhamal	Kalahandi	Total
Starchy staples	0.9193023	3.220165	0.4032135	1.356426	2.643831
Leafy vegetables	0.2217154	0.2833482	0.3658119	0.5167308	0.4386286
Vegetables	1.526038	1.41584	1.038162	1.3213	1.75425
Fruits	3.551272	2.355362	7.488017	7.098375	5.884268
Dairy	1.885066	0.5544593	3.013018	0.66061	2.004322
Meat, fish, egg	2.622052	4.423912	6.867536	5.18693	7.000006
Pulses	0.3050466	0.5234198	0.6193582	0.9600151	1.190189
Oils	1.907706	2.864471	2.071516	1.646163	2.708556
Sugar/Jaggery	0.064818	0.0656507	0.0396958	0.1566432	0.2145659
Total	6.702464	9.046818	8.395348	7.959503	8.075549

## Appendix 2

Household reliance on local markets for food from household-level primary data from TCI- TARINA.

Fig. A2 shows the proportion of households that reported having purchased a given food group in the previous 7 days.

Note: Vertical line demarcates annual average from monthly data.

Household purchase behavior by itself however tells us little about the quantities being purchased. It is not necessary that a food group being purchased by a higher proportion of households will also be purchased in a greater quantity. We therefore look at actual quantities purchased by the household over a 10- month period. While nearly all households reported purchasing vegetables, oils and protein foods, the average quantity purchased is low for these food groups. The least quantity purchased is for green leafy vegetables and fruits – both micronutrient rich food groups.

Note: Vertical line demarcates annual average from monthly data.

## Appendix 4

Note: *Minimum* refers to cost estimated taking into account the cheapest food items in each food group. *Average* refers to cost estimates taking account the average price of all food items in each food group. Prices are adjusted for edible portions. Vertical line demarcates annual average from monthly data.

Source: Primary data for prices, EAT Lancet average quantities from Willet et al. (2019).

## Appendix 5

Using primary data on food expenditures from 3600 households we find that the cost of dietary intake based on actual food purchases was $0.90 per person per day in May 2019. This falls short of the estimated cost of EAT Lancet diets by at least $2.50 per person per day (Figure A5.1).

Note: *Min* refers to cost estimated taking into account the cheapest food items in each food group. *Avg* refers to cost estimates taking account the average price of all food items in each food group. Prices are adjusted for edible portions.

Source: Primary data on expenditures and retail prices.

Note: Deficit in cost of diet is calculated as (Cost of actual food group – Cost of EAT Lancet food group). *Minimim* refers to cost estimated taking into account the cheapest food items in each food group. *Average* refers to cost estimates taking account the average price of all food items in each food group. Prices are adjusted for edible portions. N = 160. Vertical line demarcates annual average from monthly data.

Source: Primary data

*Note:* Green leafy vegetables are merged with vegetables in the CMIE averages for all- India, Bihar, Uttar Pradesh and Odisha. Vertical line demarcates annual average from monthly data.

Source: authors compilation from CPHS data

*Note:* The above data excludes spices, beverages, processed foods and foods from restaurants. The NSS data is averaged for 2011–12. The CPHS data is averaged June 2018–July 2019.

*Note:* The above data excludes spices, beverages, processed foods and foods from restaurants. The NSS data is averaged for 2011–12. The CPHS data is averaged June 2018–July 2019.

## Footnotes

2 It can adequately meet the energy requirements of a 30-year-old male (weighing 70 kg) or female (weighing 60 kg) with a moderate to high level of physical activity. The EAT Lancet dietary guidelines constitute “win-win diets” i.e. diets that fall within the safe operating space for food systems and meet the joint objectives of human health and environmental sustainability (Willet et al., 2019).

3 ICP 2017 detailed table of results: Select ‘Expenditure per capita, PPP- based (USD)’ on the website with the metadata and click on the country name for details of data source. Available here: https://databank.worldbank.org/embed/ICP-2017-Cycle/id/4add74e?inf=n.

4 The recent SOFI report does contain country-level estimates for cost of healthy diets. Those however are based on national food based dietary guidelines. For the EAT Lancet recommendations the report presents global estimates only.

5 The survey is conducted in all States and Union Territories of India except Andaman Nicobar Islands, Arunachal Pradesh, Dadra Nagar Haveli, Daman Diu, Lakshadweep, Manipur, Mizoram and Nagaland and rural region of Chandigarh. Regions not covered by the survey are essentially the north-eastern states and small islands because of their inaccessibility and/or because of their small size.

6 Excel file downloaded from https://datacatalog.worldbank.org/dataset/international-comparison-program-2017.

7 Data on portions and weights available for download here: https://www.ars.usda.gov/northeast-area/beltsville-md-bhnrc/beltsville-human-nutrition-research-center/food-surveys-research-group/docs/fndds-download-databases/.

8 Available for download here: http://www.fao.org/3/ap815e/ap815e.pdf.

9 https://data.oecd.org/conversion/purchasing-power-parities-ppp.htm.

10 Planning Commission, Government of India.

11 The cost of a healthy adequate diet is $3.75 and $3.41 per person per day, globally and for India respectively. The cost of a nutrient adequate diet is $2.33 (globally) and $1.90 (India) per person per day.
